# SECURES-Met: A European meteorological data set suitable for electricity modelling applications

**DOI:** 10.1038/s41597-023-02494-4

**Published:** 2023-09-07

**Authors:** Herbert Formayer, Imran Nadeem, David Leidinger, Philipp Maier, Franziska Schöniger, Demet Suna, Gustav Resch, Gerhard Totschnig, Fabian Lehner

**Affiliations:** 1 Institute of Meteorology and Climatology, University of Natural Resources and Life Sciences, Vienna, Austria; 2 International Water Management Institute, Lahore, Pakistan; 3 Energy Economics Group, Technische Universität Wien, Vienna, Austria; 4 Center for Energy, AIT Austrian Institute of Technology, Vienna, Austria

**Keywords:** Climate-change impacts, Energy supply and demand

## Abstract

The modelling of electricity production and demand requires highly specific and comprehensive meteorological data. One challenge is the high temporal frequency as electricity production and demand modelling typically is done with hourly data. On the other side the European electricity market is highly connected, so that a pure country-based modelling is not expedient and at least the whole European Union (EU) area has to be considered. Additionally, the spatial resolution of the data set must be able to represent the thermal conditions, which requires high spatial resolution at least in mountainous regions. All these requirements lead to huge data amounts for historic observations and even more for climate change projections for the whole 21^st^ century. Thus, we have developed the aggregated European wide climate data set *SECURES-Met* that has a temporal resolution of one hour, covers the whole EU area and other selected European countries, has a reasonable size but considers the high spatial variability.

## Background & Summary

Generally, energy system analyses in research and for system optimization have been based on one year simulations, where the weather input from selected years, representing specific climatological criteria, have been used. Due do the increasing impacts of anthropogenic climate change^1^ and the growing importance of renewable energy, an adequate representation of weather related components in energy system analyses and modelling is needed and even required by recent European regulations^2^. Up to now adequate meteorological data sets are only available for local application as for example in Murcia^3^ or Brazil^4^, but not for several countries or the European Union.

**Fig. 1:**
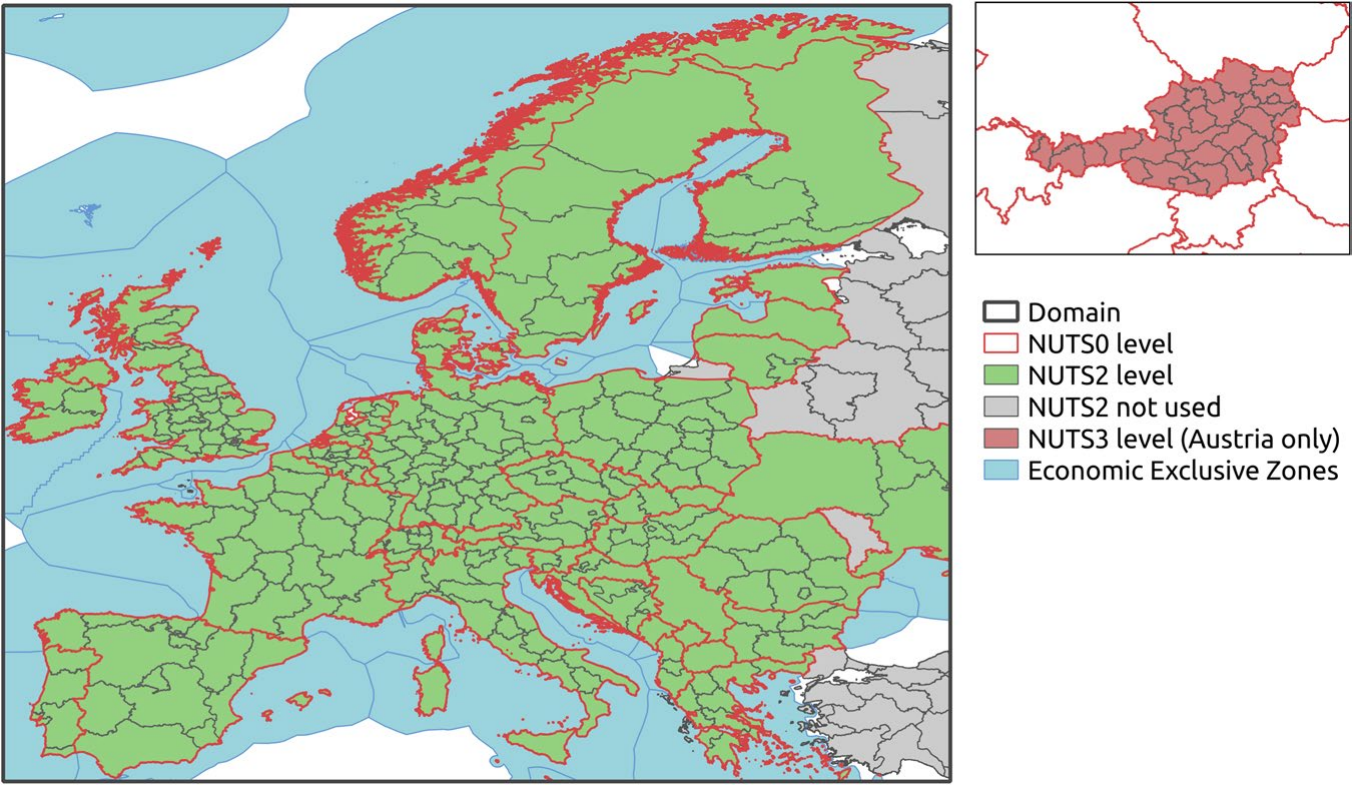
SECURES domain (green area), and the different aggregation levels used for Europe and Austria.

Within the Austrian research project “SECURES”^5^, a research consortia with experts in energy modelling, meteorology and regional climate modelling, developed a new meteorological data set (SECURES-Met)^6^, that allows a transient electricity modelling for multiple historical years and even climate change projections for the whole 21^st^ century. This European data set is quite novel as it comprises the majority of needs arising from energy modelling, without losing the necessary accuracy to represent the spatial complexity of meteorological variables. The data set has a temporal resolution of one hour and covers the continental EU electricity market (see Fig. 1), includes all relevant meteorological variables or derived indicators (see Table 2), accounts for meteorological spatial variability up to a resolution of 1 × 1 km, and data size is small enough for use in standard electricity modelling software.

To achieve all these characteristics, variable optimized aggregation algorithms have been developed and the final data sets for electricity modelling are aggregated on NUTS-0^7^ and NUTS-2 level for Europe, and for Austria additionally an aggregation on NUTS-3 level is available. Fig. 1 shows the domain of the SECURES-Met data set (green coloured area). The NUTS-0 level (states) are outlined in red and NUTS2 level (provinces) are outlined in grey. The NUTS-3 regions for Austria are shown in the Austrian map (top right). To account for the offshore wind power production, “Economic Exclusive Zones”^8^ are used (blue lines and shaded area). The aggregation of the variables and indicators to the NUTS- levels reduces the size of the data set dramatically, the data can simply be stored in ASCII format (csv files) and no compression is required.

SECURES-Met includes the air temperature at 2m-height. This allows the modelling of heating and cooling demand, the efficiency of thermal power plants (e.g., nuclear), and other temperature related processes. To model photovoltaic energy production, solar radiation flux on horizontal surface (global radiation) and the direct normal irradiance (BNI), which gives the radiation flux perpendicular to the incoming solar radiation, is available. As indicator for wind power production the “Potential Wind Power” is provided. This indicator is based on the ten-metre-wind speed, but is converted to the wind speed in 150 m above the ground to account for the height of wind power plants and multiplied with the power curve of selected off- and onshore turbines. The hydro-power indicator is based on run-off-river data and is combined with the installed potential production capacity of hydropower plants (run-off-river and reservoir) based on^9^. The hydropower indicator is only available on daily basis while all others indicators are available on hourly basis.

## Methods

The SECURES-Met dataset is based on a variety of data sets reaching from meteorological time series from reanalyses models (ERA5, ERA5-Land, COSMO-REA6) and climate change projections from regional climate models (EURO-CORDEX^10^), satellite observations (Helio-Clim), hydrological river runoff, to static data sets as the digital elevation model, NUTS regions, and population density. In Table 1 a brief description of all used data sets is shown.

**Table 1:** List of base datasets used for producing SECURES-Met.

Data Set	Type	Spatial resolution	Period
ERA5^31,32^	Reanalysis model (meteorological variables, global)	0.25° (~28 km)	1940 - present
ERA5-Land^33,34^	Reanalysis model localized (meteorological variables on land surfaces, global)	0.1° (~11 km)	1950 - present
COSMO-REA6^18,35^	High resolution reanalysis (wind speed, Europe)	0.055° (~6 km)	1995–2019
ICHEC-EC-EARTH-rcp45-rlilp1_KNMI-RACMO22E^10^	Regional climate model scenario (meteorological variables, Europe, low emission scenario)	0.11° (~12 km)	1951–2100
ICHEC-EC-EARTH-rcp85-rlilp1_KNMI-RACMO22E^10^	Regional climate model scenario (meteorological variables, Europe, high emission scenario)	0.11° (~12 km)	1951–2100
HelioClim^30,36^	Satellite observation (Radiation variables, Europe)	~4–5 km	2004 - present
E-Hype^37,38^	Regional climate model scenarios (river flow, Europe)	~5 km	1951–2100
JRC hydropower plant data base^39,40^	Hydropower database (Europe)	Point	2019
LsPop 2008^41^	Population density (global)	1 km	2008
NUTS^7^	Administrative units of Europe	State, basic and small regions	1995
DEM^42^	Digital Elevation Model of Europe	1 km	2023
EEZ^8^	Economic Exclusive Zone of Europe	Basic regions	2023

The table includes the name of the dataset, references, short description, spatial resolution and the period the data set covers.

The meteorological variables are based on historical observations taken from the ERA5-Land reanalysis data set. This data set contains all necessary meteorological variables on land on hourly basis, with a spatial resolution of 0.1° (~11 km). For offshore areas (Exclusive Economic Zone, EEZ) the ERA5 data has been rescaled to the ERA5-Land grid using a patch regridding method from ESMF^11^. This was also done for the climate scenarios to have all meteorological information on the same grid and same projection.

The selected climate change projections reflect the possible range of human behaviour in terms of greenhouse gas emissions within the 21^st^ century. During a stakeholder process within the SECURES project, it was decided, to have one low emission scenario that fulfils the two-degree criteria from the UNFCCC Paris agreement from 2015. The second projection should be a high emission scenario usually described as “business as usual”. The selection of the models is based on the ensemble analyses of the newest climate model generation CMIP6, where the emission scenario SSP1-2.6 fulfils the two-degree target and the scenario SSP3-7.0 is taken as business as usual. The selected EURO-CORDEX models were forced by the RCP 4.5 for the low emission scenario and by RCP 8.5 for the high emission scenario.

The selection of possible regional climate projections was limited to the models, for which hydrological river runoff data for whole Europe on daily basis were available. The E-HYPE database provides such information. For the low emission scenario we selected the model “ICHEC-EC.EARTH-rcp45-rlilp1_KNMI-RACMO22E” and for business as usual “ICHEC-EC.EARTH-rcp85-rlilp1_KNMI-RACMO22E”. In a later section, an evaluation how well these models fulfil the representation of the ensemble mean is shown (see Fig. 4). All meteorological variables used from these two models were bias corrected with the ERA5-Land data with a quantile delta mapping approach^12^.

As the climate change projections only provide daily data, a temporal disaggregation was required. For that purpose, the surface wind and global radiation were disaggregated using a statistical approach. Historical hourly ERA5 and ERA5-Land (1991–2020) data were used to build an average day-curve for every day of the year. This curve was further smoothed by applying a seven-day rolling mean for every hour individually. To ensure continuous data at day changes, the mean values of two consecutive days were averaged during the hours near day changes. Temperature was disaggregated by modelling altering minimum and maximum temperatures of consecutive days with a cosine function, following^13^. For that purpose, the declination was calculated with the methods of Bourges^14^ and Spencer^15^. The minimum temperature was assumed to occur at sunrise rounded to the full hour, the maximal temperature two hours after noon, also rounded to the full hour.

A core task for the data generation was to minimize the data set size but conserving as much as possible of the necessary high spatial resolution information. So for every indicator, an optimized disaggregation to gain high spatial resolution information and an aggregation to reduce data size was developed.

For temperature and radiation data, those variables are specifically relevant in locations with high population density and therefore high electricity demands. Where people live, it is more likely for solar panels to be set up on roofs. For temperatures in mountainous area, a high spatial resolution is crucial, as temperature has a strong elevation dependency and the population density is higher at the low elevated valleys. Therefore, the two-metre-temperature and the global radiation were calculated as

- areal mean, where every grid cell was weighted an equal amount,
- and population density weighted mean, where the LsPop population density data was used on a one-kilometre basis during aggregation.

Radiation was provided in W/m² as two parameters. First, as the global radiation (GLO) directly available from ERA5-Land and the EURO-CORDEX models. Second, the direct normal irradiance (BNI), which is the radiation on a surface normal to the direction of the sun, was calculated. This parameter is required to calculate the incident radiation on an inclined surface, for example a solar panel. The HelioClim dataset was used to estimate BNI from global radiation for every NUTS-2 region for each hour of the day. The estimation was done using quantile mapping on an eight-day basis. GLO values below 10 W/m^2^ were not converted to BNI, but GLO was taken directly. This approach was chosen due to lacking data quality for low radiation. BNI exceeding the solar constant of 1361 W/m² were set to that value. HelioClim data have limitations at latitudes higher than 60 degree. At latitudes above 60 °C (mainly Scandinavia), GLO is a more robust radiation indicator.

**Fig. 2:**
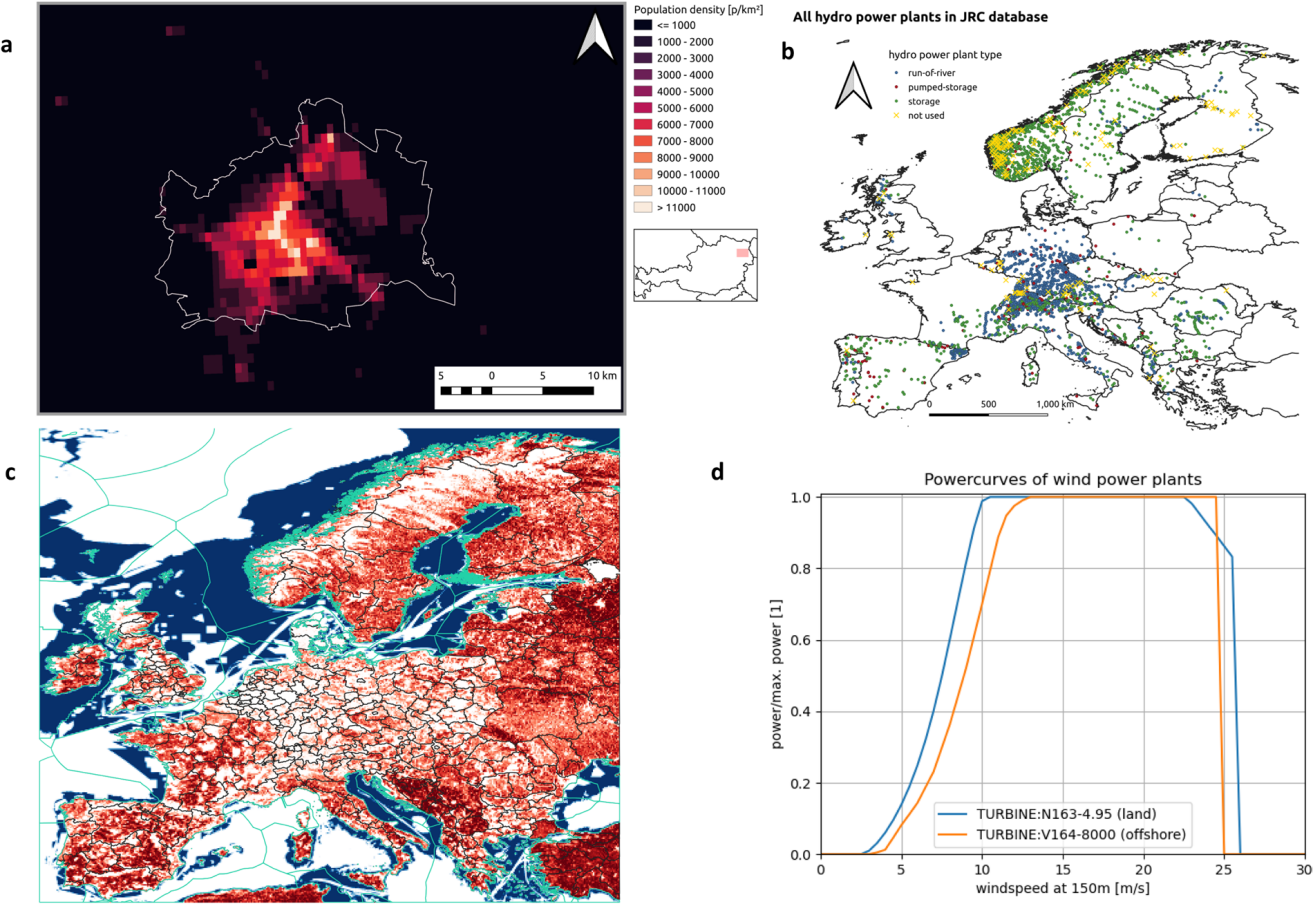
Population density for the City of Vienna, Austria as example for the LsPop dataset (**a**), locations of the hydropower plants from JRC database (**b**), wind mask for land (red) and offshore (blue). The darker the red colour the higher is the fraction within an ERA5-Land grid cell, that is suitable for wind power plants. White indicates, that no wind power plants are allowed (**c**). Relative power curves as a function of wind speed at 150 m above ground used for land (blue) and offshore (orange) wind power plants.

As temperature is highly dependent on altitude, a lapse rate of –6.5 °C/km was applied for the disaggregation from the ERA5-Land grid to the 1 km grid of LsPop for every individual hourly value. Temperature was provided in °Celsius. Fig. 2a shows an example for the high spatial resolution of the LsPop data set and Fig. 6a a comparison of the spatial mean and population weighting aggregation in the period 1981–2010 as an example for the differences. Population weighted temperature tends to be warmer because valleys, where people live, are weighted higher in mountainous regions and thus the temperature is more accurate than a simple spatial mean.

For the wind power indicator several steps have been necessary. As the turbine height of wind power plants is approximately 150 m above ground level and the ERA5(-Land) and EURO-CORDEX variables provide wind speed at 10 m height, wind speeds had to be converted to the higher altitudes. This transfer is not linear and depends on roughness, vertical stability and topography. Therefore a statistical approach was chosen. The COSMO-REA6 dataset provides wind speed at 150 m and the period of 1995–2019. This data was regridded to the ERA5-Land resolution. Afterwards, the empirical cumulative distribution function was calculated for each grid point and the 10 m surface wind percentiles of the models were mapped to the percentiles of the COSMO-REA6 to get the corresponding wind speed at 150 m. Then the relative power curves for land and offshore wind turbines, visible in Fig. 2d were applied to the wind speed. As representative turbines N163-4.95^16^ was chosen for land and V164-8000^17^ for offshore. Afterwards, a map provided by COSMO-REA6^18^, indicating suitable spots for wind power plants across Europe was aggregated to the ERA5-Land grid by building the arithmetic mean, yielding the fraction of suitable area per grid box. This mask was then used as weights for the power curves to aggregate the wind power to the NUTS regions, and EEZ and is also visible in Fig. 2c. The power output is therefore normalized to 1. Efficiency loss was not considered but can be applied by users of the data set using their own assumptions.

The hydropower indicator is based on the E-HYPE scenarios. The utilized variable is the daily mean river discharge in m³/s. As river discharge in Europe has no distinct diurnal cycle, disregarding very small highly glaciated catchments in summer, river discharge is not subject to change on hourly basis, daily resolution is sufficient and no temporal disaggregation was required.

The JRC Hydro Power Plant Database was used for information and characteristics about the hydropower plants over Europe. Missing data of average annual generation was estimated using the representative full load hour (FLH) for hydropower from TYNDP 2020 scenarios from ENTSO-E^9^ by considering distributed generation scenario and climate of the year 1984 in the specific countries.

Individual power plants were first divided into runoff-river and reservoir plants and then attributed to a specific E-HYPE sub-basin. The daily mean power was then assumed to be proportional to the daily mean runoff, up to a maximum capacity for run-of-river power plants. The scaling factor between daily mean discharge and daily mean power was calculated from the mean annual energy production of each individual plant in an iterative process. Afterwards, the results in MW were normalized to 1 by dividing through the annual mean power.

As every data set, also SECURES-Met^6^ has limitations, stemming from the data sets used or the methods deployed to produce the final product. Some information concerning skills and limitation of the data sets used, can be found in the references listed in Table 1. An evaluation of ERA5 and ERA5-Land is given in^19^. Concerning wind data, an evaluation of ERA 5 Land and COSMO-REA5 is given in^20^.Base quality information concerning E-hype can be found in^21^ and an evaluation of EURO-CORDEX regional climate models with the focus on precipitation is given in^22^. The population density data used for aggregation is from the year 2008 and a more recent data set (e.g. GPWv4^23^) would be more accurate, but the effect on the aggregation should be minimal. The used constant temperature lapse rate is also a simplification, that especially leads to an overestimation of the lapse rate during winter and night-time temperatures due to inversions, but it is still an improvement compared to the coarse resolution data in complex terrain.

## Data Records

SECURES-Met^6^ is available in a tabular csv format for the historical period (1981–2020, hydro power only until 2010) created from ERA5 and ERA5-Land and two future emission scenarios (RCP 4.5 and RCP 8.5, both 1951–2100, wind power starting from 1981, hydro power from 1971) created from one CMIP5 EURO-CORDEX model (GCM: ICHEC-EC-EARTH, RCM: KNMI-RACMO22E) on the spatial aggregation level

NUTS0 (country-wide),

NUTS2 (province-wide),

NUTS3 (province-Austria only),

and EEZ (Exclusive Economic Zones, offshore only).

The data is divided into the historical (Historical.zip) and the two emission scenarios (Future_RCP45.zip and Future_RCP85.zip), a README file, which describes, how the files are organized, and a folder (Meta.zip), which has information and shape files of the different NUTS levels. As population weighted temperature and radiation represent values in geographical complex areas in a more representative way for solar power, it is highly recommended to use population weighted files. Spatial mean should be used for reference only.

Data can be downloaded as zip files. Each zip (except Meta.zip) is organized in the following directory structure: AGGREGATION-LEVEL/DATAFILE.csv

AGGREGATION-LEVEL is one of the following:

NUTS0_Europe aggregated on NUTS0 level (countries) for Europe

NUTS2_Europe aggregated on NUTS2 level (provinces) for Europe

NUTS3_Austria aggregated on NUTS3 level (districts) for Austria

NUTS0_offshore aggregated on NUTS0 level (countries) for Europe, offshore, wind power only

EEZ_offshore aggregated on EEZ level (economic exclusive zones) for Europe, offshore, wind power only

DATAFILE is composed like: VARIABLE_AGGREGATION-LEVEL_REGION_AGGREGATION-METHOD_MODEL_FREQUENCY_TIMEPERIOD

VARIABLE is one of:

HYD-RES daily mean power from reservoir plants, unit: MW (sum aggregation), 1 (normalized aggregation)

HYD-ROR daily mean power from ROR plants, unit: MW (sum aggregation), 1 (normalized aggregation)

GLO hourly mean global radiation, unit: W*m**-2

BNI hourly direct normal irradiation, unit: W*m**-2

T2M hourly air temperature 2 m above ground, unit: °C

WP hourly potential wind power production, normalized between 0 and 1, unit: 1

REGION is one of: offshore, Europe and Austria

**Fig. 3:**
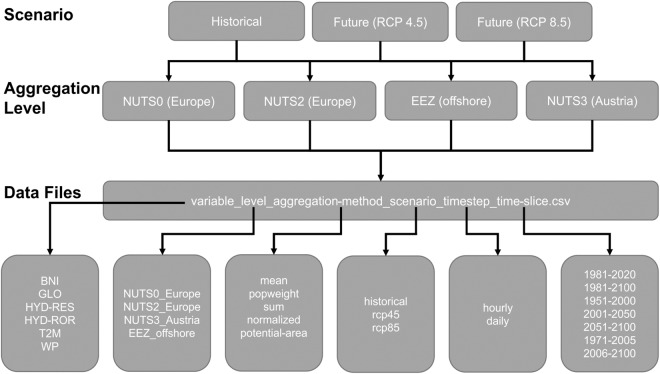
Organigram of the file structure and the information given by the file name of the SECURES-Met data set.

In Fig. 3 an organigram of the structure of the SECURES-Met data is shown. All data are open source and available at ZENODO in the same structure.

In Table 2 all final products of SECURES-Met are described shortly.

**Table 2:** List of variables and definitions within the SECURES-Met data set.

Variable	Short name	Unit	Aggregation methods	Temporal resolution
Temperature (2 m)	T2M	°C	spatial mean	hourly
		°C	population weighted mean	
Radiation	GLO (mean global radiation)	Wm-2	spatial mean/population weighted mean	hourly
	BNI (direct normal irradiation)	Wm-2	spatial mean/population weighted mean	
Potential Wind Power	WP	1	normalized with potentially available area and power curve	hourly
Hydro Power Potential	HYD-RES (reservoir)	MW	summed power production	daily
	HYD-ROR (run-of-river)	1	summed power production normalized with average daily production	

All these products are available for historical period from 1981 till 2020 and for two climate change projections, one low emission scenario fulfilling the 2 degree target^24^, and a high emission scenario that follow the CMIP 6^25^ recommendation for “business as usual” scenario SSP3-7.0^26^, for the whole 21^st^ century.

All data is available at Zenodo^6^

## Technical Validation

**Fig. 4:**
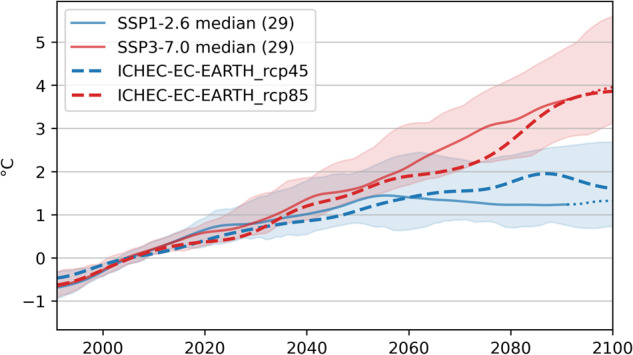
Ensemble median (line) and 10^th^ to 90^th^ percentile range (coloured area) of the annual mean temperature anomaly compared to current climate (1991–2020) of the SECURES-Met Europe domain, for the CMIP6 ensemble forced by the SSP1-2.6 (blue) and SSP3-7.0 (red) emission scenario. The number in the parenthesis indicates the numbers of models within the ensemble. The dashed lines gives the same indicator for the two selected EURO-CORDEX models.

The two selected regional climate projections represent the average development of climate scenarios fulfilling the two-degree target (low emission scenario) and the business as usual scenario (high emission scenario). In Fig. 4 the development of the annual mean temperature of the two selected models (dashed blue and red lines) during the whole 21^st^ century and the European domain is compared with the ensemble (29 models) of global climate models of the newest generation (CMIP6) for the low emission scenario SSP1-2.6 (blue) and the high emission scenario SSP3.7.0 (red). The bold lines represent the ensemble medians and the shaded areas the range between the 10^th^ and 90^th^ percentile. Both selected models lie within the 10^th^ and 90^th^ percentile of the respective ensemble during the 21^st^ century and for the most time of the century, the difference between the selected model and the ensemble median of the ensemble is in the order of few 0.1 °C and thus much smaller than the climate change signal. The two selected models are therefore good representatives for the ensemble means of the selected emission scenarios, especially till the middle and at the end of the 21^st^ century.

**Fig. 5:**
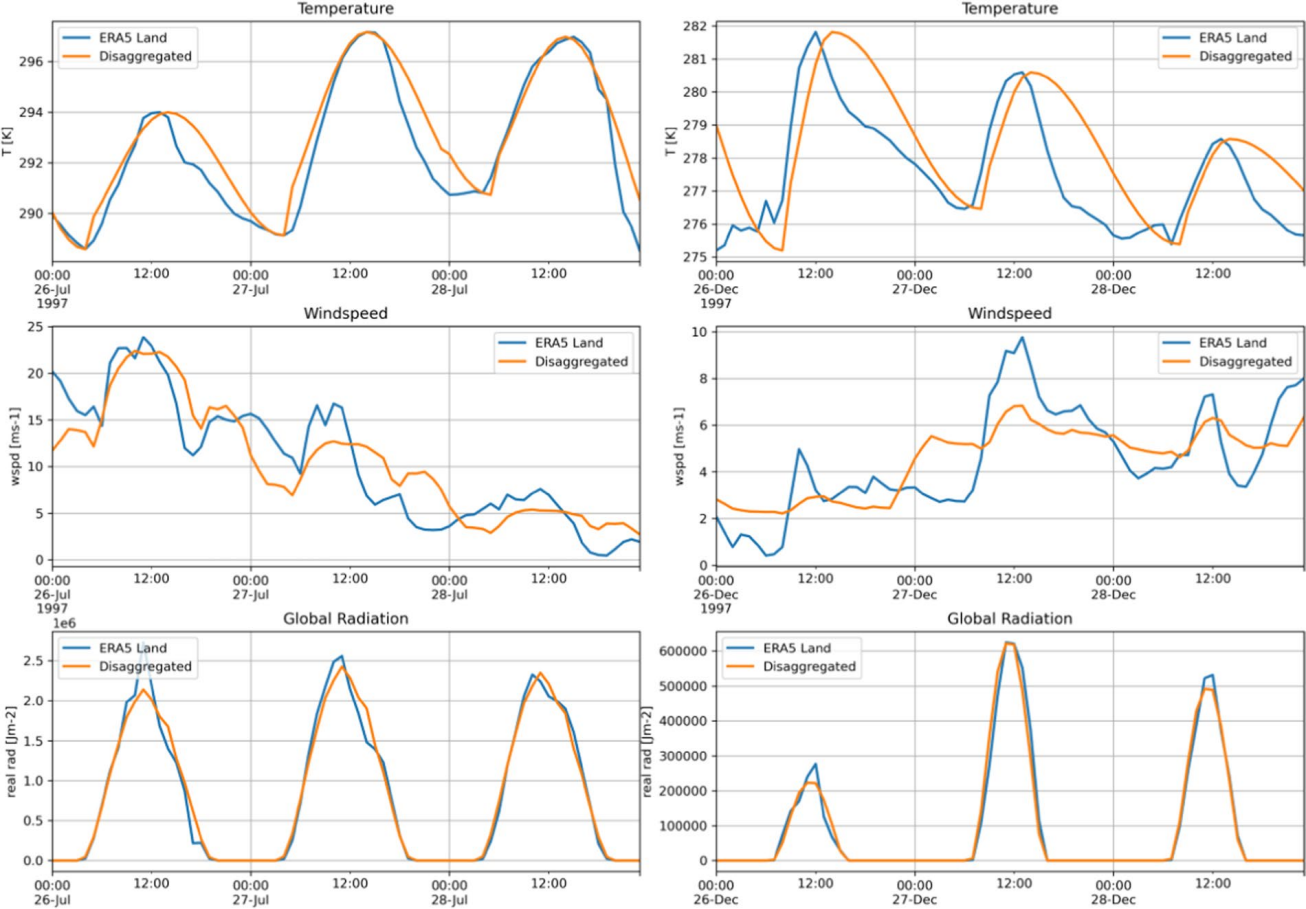
Comparison of hourly values for all necessary meteorological variables from ERA5-Land (blue) and the temporal disaggregated values estimated from daily data (orange) for a grid cell near Vienna, Austria. The left side represents a summer case and the right side a winter case.

The quality of the temporal disaggregation of daily meteorological data to hourly data is shown in Fig. 5. For this comparison, the ERA5-Land hourly values are aggregated to daily values, as they are provided by regional climate projections, subsequently disaggregated with the selected statistical method and then compared with the original hourly data. The results show a good reproduction of the diurnal cycle for all three variables in summer cases as well as winter cases.

**Fig. 6:**
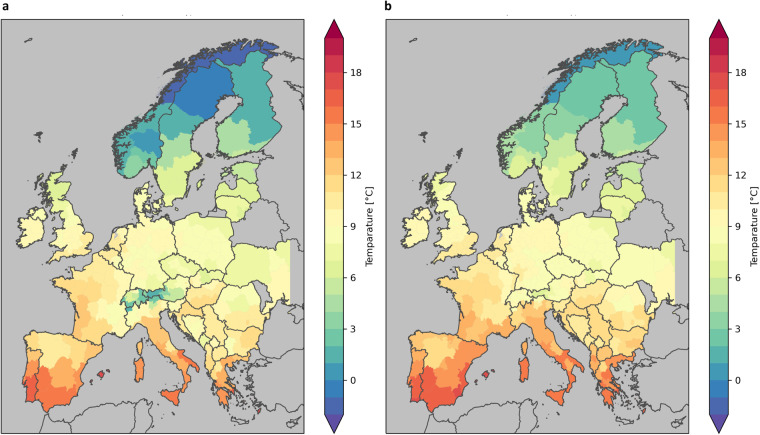
Comparison of temperature aggregation methods. Spatial mean (**a**) and population weighted (**b**) annual mean temperature for the period 1981–2010 [°C]. In mountainous areas, the population weighted temperature is higher, as the majority of people live in the low elevated valleys.

In Fig. 6 comparison of the climatological mean temperature for the period 1981–2010 for the whole SECURES-Met domain is shown, based on simple arithmetic averaging for the NUTS2 region (**a**) and population weighted average (**b**). In flat areas (e.g. northern Germany or Poland), insignificant differences occur but in mountainous areas (e.g. Austria or Norway), the population weighted mean temperature is significantly warmer. This represents the real heating and cooling demand of the people living in mountainous regions much better than a simple arithmetic mean.

**Fig. 7:**
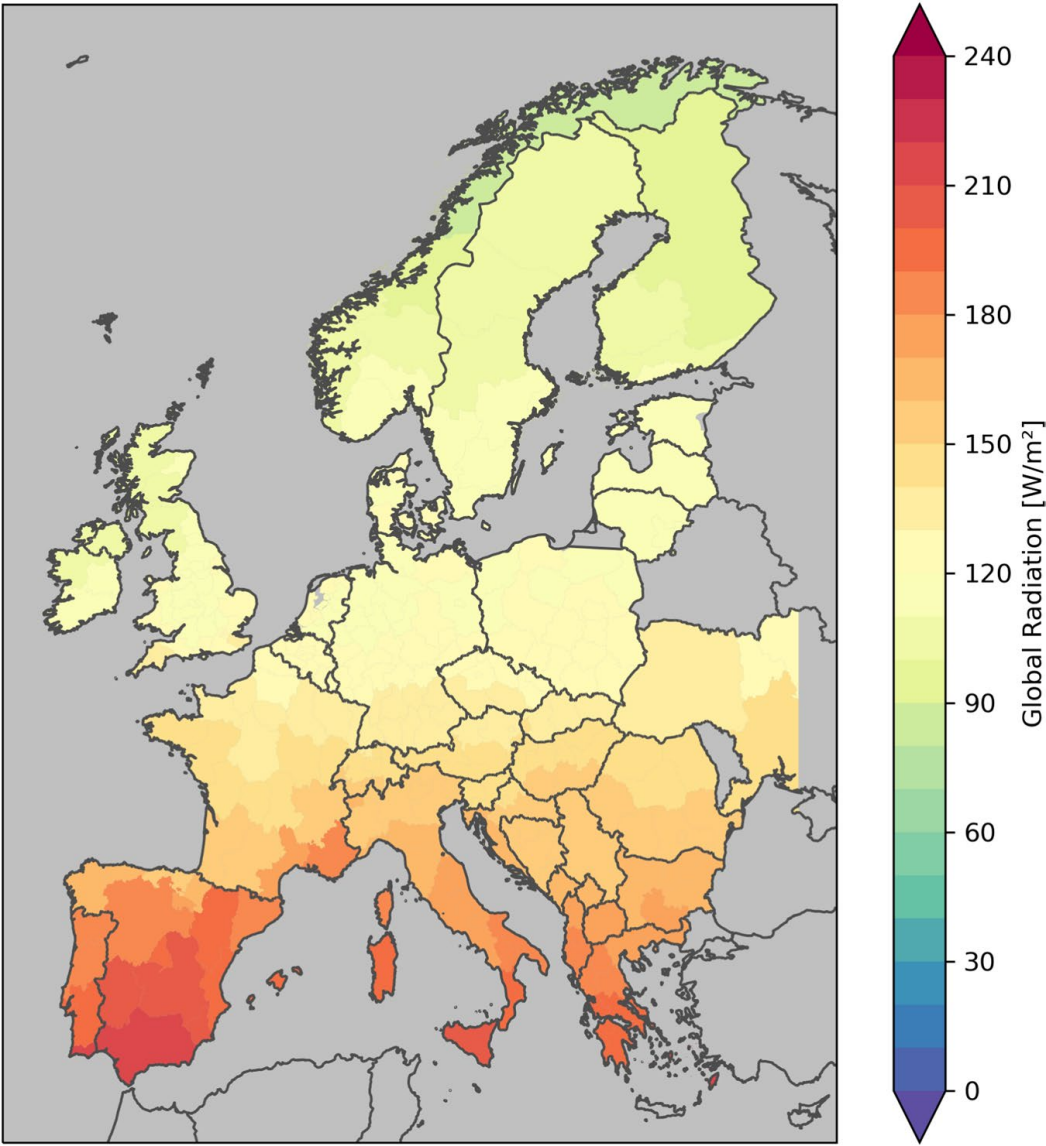
Radiation climatology for the period 1981–2010 for SECURES-Met land domain, based on ERA5-Land for global radiation. Aggregated population weighted on NUTS 2 regions.

For radiation, also the population weighted mean is better representing the real situation, as photovoltaic panels frequently are mounted on roofs. In Fig. 7 the climatological mean of the variable GLO is shown. The variable BMI is similar, both variables reflect the north-south gradient and BNI is systematic higher than GLO, but the range is modified by different cloudiness in Europe.

**Fig. 8:**
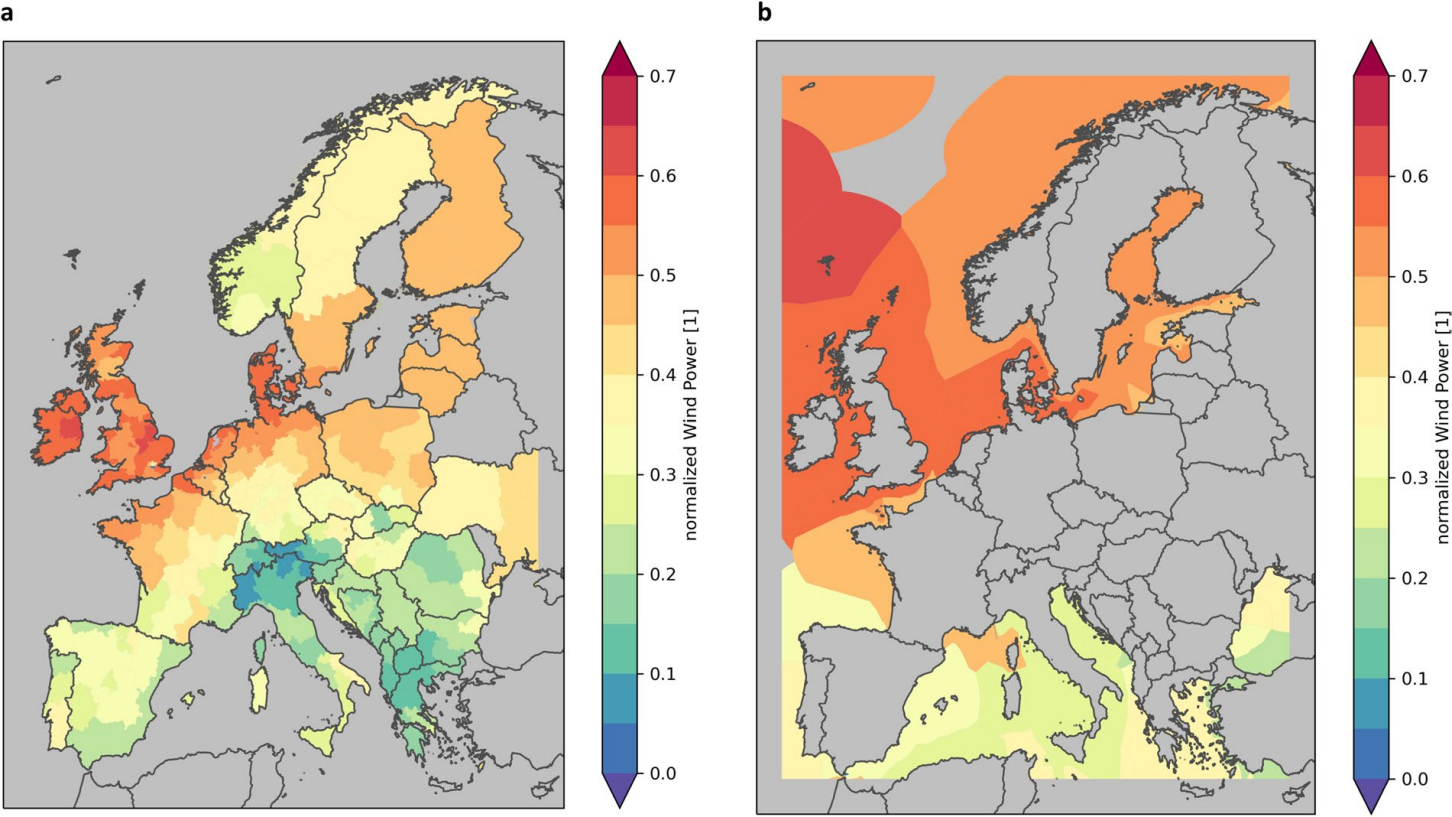
Normalized wind power climatology for the period 1981–2010 for SECURES-Met land domain, based on ERA5-Land (**a**) and for SECURES-Met offshore regions, based on ERA5 (**b**). Aggregated on NUTS-2 regions on land and on EEZ regions.

In Fig. 8 the normalized wind power climatology is shown for NUTS-2 on land and for EEZ on the ocean. The wind power potential is not only depending on the local wind speed but also on the area suitable for wind power plants. This is clearly seen in Norway. Here the offshore locations have high wind power potential and the land area, due to the mountainous character, only low.

**Fig. 9:**
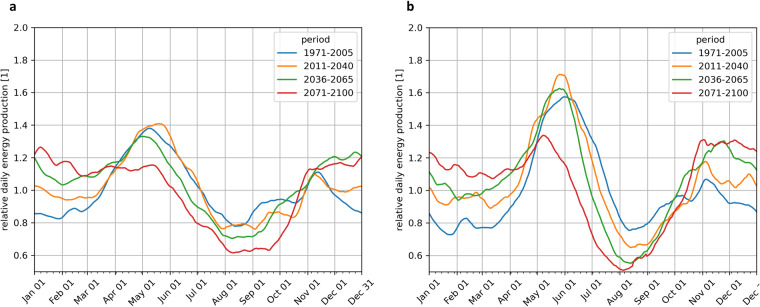
Annual course of energy production for run-of-river (**a**) and reservoir (**b**) hydropower plants for the RCP 8.5 scenario, normalized to 1 with the mean energy production of the period (1971–2005) in Austria.

To validate the reliability of the hydropower generation, the seasonal cycle for the run-off-river plants (Fig. 9a) and the reservoir plants (b) are shown for historical production (blue line) in Austria. Within the current climate the run-off-river plants have two maxima, one in April/May and one in November, whereas the spring maxima is associated with the peak of snow melt in the mountains. This maximum vanishes in the business as usual scenario till the end of the 21^st^ century and only one maximum can be seen during winter. The reservoir plant production has a clear maximum during the snow and glacier melt period from May to July in the current climate. Till the end of the century (red line) this maximum weakens significantly and occurs earlier in the year and a pronounced reduction occurs during summer. These findings correspond well with common results of hydrological scenarios for Europe in the 21^st^ century^27^.

## User Notes

This dataset provides the basis for direct use or further processing in energy system modelling. The generated time series for wind (onshore and offshore) and hydropower generation (run-of-river and reservoir) can be directly used in energy system modelling. The hydro power data are only given on daily base, as run–of-river hydro power generation has no distinct diurnal cycle and reservoir hydro power generation is strongly controlled by demand. As the unit for this indicator is power (unit Megawatt), the daily values can be used as hourly values if needed. This implies that the hydro power generation has no diurnal cycle. In most energy system models daily or even weekly or monthly representation of hydro power operation is used^28^.

Temperature and radiation data can be used to model solar energy production and temperature-dependent electricity demand and supply components like heating and cooling demand, efficiency losses of batteries or photovoltaic panels, and thermal losses of thermal power plants. This can be used to assess security of supply in the context of climate-related extreme events in electricity systems or adequacy problems in future energy systems.

An exemplary application of the dataset can be found in further publications in the course of the project SECURES (see https://www.secures.at/publications).

## Data Availability

The data are stored as ASCII text (csv) and no specific software is necessary to access the data. The production of the data was done with Python. These scripts are mainly data manipulation routines and do not contribute in processing the data further. To assure repeatability all scripts are available at Zenodo (10.5281/zenodo.8108927)^29^.

## References

[1] Bo-Tao, Z. and Q. Jin, *Changes of weather and climate extremes in the IPCC AR6*. Advances in Climate Change Research, **17**(6): p. 713 (2021).

[2] Dubus L (2022). Towards a future-proof climate database for European energy system studies. Environmental Research Letters.

[3] Ibarrarán, M. E. *et al*. *Climate Change and Natural Disasters: Macroeconomic Performance and Distributional Impacts, in Environment, Development and Sustainability*.: London. p. pp. 549–569 (2009).

[4] Deng Y (2023). Harmonized and Open Energy Dataset for Modeling a Highly Renewable Brazilian Power System. Scientific Data.

[5] Schöniger, F. *SECURES - Securing Austria’s Electricity Supply in Times of Climate Change*. [cited 2023; Available from: https://www.secures.at/.

[6] Formayer H (2023). Zenodo.

[7] Eurostat, N. *Nomenclature of territorial units for statistics*. Eurostat: Luxembourg, 1995.

[8] Treves, T. *United Nations Convention on the Law of the sea*. United Nations Audiovisual Library of International Law (http://untreaty.un.org/cod/avl/pdf/ha/uncls/uncls_e.pdf) (2008).

[9] TYNDP2020, *TYNDP2020 Scenario Data Sets Distributed Energy scenario, 1984 climate year*, in (2020).

[10] Jacob D (2014). EURO-CORDEX: new high-resolution climate change projections for European impact research. Regional Environmental Change.

[11] Zhuang, J. *xESMF Documentation*. (2019).

[12] Lehner F, Nadeem I, Formayer H (2023). Evaluating skills and issues of quantile-based bias adjustment for climate change scenarios. Advances in Statistical Climatology. Meteorology and Oceanography.

[13] Förster K (2016). An open-source MEteoroLOgical observation time series DISaggregation Tool (MELODIST v0. 1.1). Geoscientific Model Development.

[14] Bourges B (1985). Statistical Distribution of Solar Radiation: A European Data Set of Cumulative Frequency Curves of Solar Irradiance on Tilted Planes. International Journal of Solar Energy.

[15] Spencer JW (1971). Fourier Series Representation of the Position of the Sun. Search.

[16] *N163/5.X*. Available from: https://www.nordex-online.com/de/product/n163-5x/.

[17] *Vestas*. Available from: https://en.wind-turbine-models.com/turbines/318-vestas-v164-8.0.

[18] Frank CW (2020). The added value of high resolution regional reanalyses for wind power applications. Renewable Energy.

[19] Valipour M, Dietrich J (2022). Developing ensemble mean models of satellite remote sensing, climate reanalysis, and land surface models. Theoretical and Applied Climatology.

[20] Jourdier B (2020). Evaluation of ERA5, MERRA-2, COSMO-REA6, NEWA and AROME to simulate wind power production over France. Adv. Sci. Res..

[21] Lindström G (2010). Development and testing of the HYPE (Hydrological Predictions for the Environment) water quality model for different spatial scales. Hydrology research.

[22] Prein A (2016). Precipitation in the EURO-CORDEX 0.11° 0. 11° and 0.44° 0. 44° simulations: high resolution, high benefits?. Climate dynamics.

[23] Center for International Earth Science Information Network, C.C.U., *Gridded Population of the World, Version 4 (GPWv4): Population Density, Revision 11*. 2018, NASA Socioeconomic Data and Applications Center (SEDAC): Palisades, New York.

[24] Rogelj J (2016). Paris Agreement climate proposals need a boost to keep warming well below 2 C. Nature.

[25] Eyring V (2016). Overview of the Coupled Model Intercomparison Project Phase 6 (CMIP6) experimental design and organization. Geoscientific Model Development (Online).

[26] Riahi K (2017). The Shared Socioeconomic Pathways and their energy, land use, and greenhouse gas emissions implications: An overview. Global environmental change.

[27] Zhao T, Dai A (2022). CMIP6 model-projected hydroclimatic and drought changes and their causes in the twenty-first century. Journal of Climate.

[28] Rheinheimer, D. E. *et al*. Hydropower representation in water and energy system models: a review of divergences and call for reconciliation. *Environmental Research: Infrastructure and Sustainability*, 2023.

[29] Formayer H, Leidinger D, Nadeem I, Maier P, Lehner F (2023). Zenodo.

[30] *HelioClim-3 solar radiation database*.; Available from: https://www.soda-pro.com/help/helioclim/helioclim-3-overview (2023).

[31] Hersbach, H. *et al*. *ERA5 hourly data on single levels from 1959 to present [Dataset]. Copernicus Climate Change Service (C3S) Climate Data Store (CDS)*. 10.24381/cds.adbb2d47.

[32] Hersbach H (2020). The ERA5 global reanalysis. Quarterly Journal of the Royal Meteorological Society.

[33] Muñoz-Sabater J (2021). ERA5-Land: A state-of-the-art global reanalysis dataset for land applications. Earth System Science Data.

[34] Muñoz Sabater, J. ERA5-Land hourly data from 1950 to present. *Copernicus Climate Change Service (C3S) Climate Data Store (CDS)*. 10.24381/cds.e2161bac (2019).

[35] Bollmeyer C (2015). Towards a high‐resolution regional reanalysis for the European CORDEX domain. Quarterly Journal of the Royal Meteorological Society.

[36] Blanc P (2011). The HelioClim project: Surface solar irradiance data for climate applications. Remote Sensing.

[37] Donnelly C, Andersson JCM, Arheimer B (2016). Using flow signatures and catchment similarities to evaluate the E-HYPE multi-basin model across Europe. Hydrological Sciences Journal.

[38] *World-Wide Hydrological Predictions*. 2023 2023 07 12]; Available from: https://hypeweb.smhi.se/.

[39] European Commission, J.R.C.J., *JRC Hydro-power database*., J.R.C.J. European Commission, Editor. (2019).

[40] Felice MD, Peronato G, Kavvadias K (2021). Zenodo.

[41] Nations, U. World population prospects: The 2008 revision population database. Department of Economics and Social Affairs, Population Division, United (2008).

[42] DEM-Europe. 2023, *European Environment Agency*.

